# Association between consumption of ultra-processed food at midlife and handgrip strength at late life: The Singapore Chinese Health Study

**DOI:** 10.1016/j.jnha.2025.100634

**Published:** 2025-07-18

**Authors:** Yue Li, Kevin Y. Chua, An Pan, Woon-Puay Koh

**Affiliations:** a Department of Epidemiology and Biostatistics, Ministry of Education Key Laboratory of Environment and Health, School of Public Health, Tongji Medical College, Huazhong University of Science and Technology, Wuhan, Hubei Province, China; b Integrative Sciences and Engineering Programme, NUS Graduate School, National University of Singapore, Singapore; c Healthy Longevity Translational Research Programme, Yong Loo Lin School of Medicine, National University of Singapore, Singapore; d A⁎STAR Institute for Human Development and Potential, Singapore

**Keywords:** Epidemiology, Food processing, Handgrip strength, Muscle strength, Ultra-processed food

## Abstract

**Objectives:**

Cohort studies with sufficient follow-up that include an assessment of overall diet quality are necessary to determine the prospective relationship between consumption of ultra-processed foods (UPFs) and muscle strength.

**Design:**

Prospective cohort study.

**Setting and participants:**

We included 13,570 participants from the Singapore Chinese Health Study who were recruited at a mean age of 53 years from 1993 to 1998, and re-visited at follow-up 3 when they were at a mean age of 74 years from 2014 to 2017, after a median follow-up of 21.2 years.

**Measurements:**

At baseline, UPFs were defined according to the Nova classification using dietary information collected via a validated 165-item food-frequency questionnaire. During the third follow-up visits, handgrip strength was measured using a digital grip strength dynamometer. Muscle weakness was defined as the lowest sex-specific quintile of handgrip strength.

**Results:**

Compared to participants in the lowest quintile of UPF consumption, those in the highest quintile of UPF consumption showed a reduction of 0.411 kg [95% confidence interval (CI), 0.148, 0.674] in absolute handgrip strength, a reduction of 0.229 kg/m (95% CI, 0.071, 0.387) in handgrip strength by height, and an OR of 1.32 (95% CI, 1.15, 1.53) for muscle weakness. These associations remained significant after adjusting for overall diet quality.

**Conclusion:**

Higher UPF consumption in midlife is linked to lower handgrip strength in later life, independent of overall diet quality.

## 1 Introduction

Population aging is a global issue contributing to increased health and economic burdens, partially due to muscle strength decline [1]. Handgrip strength is a reliable indicator of muscle strength [2], and low handgrip strength in older adults has been associated with high risks of hospital-associated disability [3], dementia [4], cardiovascular disease [5], and mortality [6]. Improving muscle strength can mitigate such risks.

Ultra-processed foods (UPFs) are industrial formulations enhanced with additives for flavor, appearance, and sweetness. Higher UPF consumption has been associated with adverse outcomes in aging, such as frailty, dementia, and mortality [[7], [8], [9]]. However, there is limited research on UPFs’ direct impact on muscle strength or sarcopenia or its components (i.e. muscle mass and strength) [[10], [11], [12]]. To our best knowledge, only one prospective study has reported that higher UPF intake, independent of healthy diet score, was associated with faster grip strength decline, but this was a study of relatively young adults at a mean age of 48 years with a short median follow-up duration of 3.0 years [13].

This study leveraged a population-based cohort of Chinese adults in Singapore with over 20 years of follow-up. We examined the association between UPF consumption in midlife and handgrip strength in late life and tested whether it was confounded by diet quality.

## 2 Methods

### 2.1 Study population

The Singapore Chinese Health Study (SCHS) is a prospective cohort in Singapore that recruited 63,257 Chinese adults who are aged 45–74 years from 1993 to 1998 [14]. Follow-up interviews were conducted in three phases: first (1999–2004), second (2006–2010), and third (2014–2017). This study was approved by the institutional review board at the National University of Singapore, and written informed consent was obtained from all study participants.

In this study, we included 13,789 participants assessed for handgrip strength at the third follow-up. After excluding participants with implausible energy intake (men with <700 kcal/day or >3700 kcal/day or women with <600 kcal/day or >3000 kcal/day) or missing height data at baseline, 13,570 participants were included in the analysis (Supplemental Figure S1).

### 2.2 Assessment of diet at midlife

At baseline, habitual dietary intakes were collected through a validated 165-item food frequency questionnaire (FFQ) [14]. The details of FFQ are described in Supplemental Method 1. Nova food classification was applied to food items in the FFQ, dividing beverage and food items into four groups according to the extent and purpose of food processing: (1) unprocessed or minimally processed foods, e.g. fresh vegetables; (2) processed culinary ingredients, e.g. salt; (3) processed foods, e.g. canned fish; (4) UPFs, e.g. processed meat [15]. Because alcohol is linked to chronic diseases [16], and the Nova classification does not classify alcoholic beverages directly, we did not include alcohol as UPF in the primary analysis. Furthermore, given that increased whole grain consumption has been linked to higher levels of successful aging [17], we did not classify whole-wheat bread as an ultra-processed food in our primary analysis. UPFs were identified in our study based on two key characteristics of food items: (1) the presence of additives that enhance palatability or appeal, and (2) the use of ingredients not commonly found in home kitchens [15]. Of the 165 items in FFQ, 26 items (15.8%) belonged to UPFs, such as soft drinks, sausage, and margarine. Details on UPFs are shown in Supplemental Table S1. For each participant, UPF intake was evaluated as the weight of UPFs (g/day, the sum of food items in the UPF group) divided by the weight of total food items (g/day). We used weight proportion (%) rather than energy ratio since the former better captures non-nutritional factors related to food processing, e.g. additives, alterations to the structure of raw foods, and neo-formed contaminants [18,19].

### 2.3 Assessment of covariates at midlife

At baseline, trained investigators collect information through a structured questionnaire. Detailed information on covariates can be found in Supplemental Method 2.

### 2.4 Assessment of handgrip strength in late life

During the third follow-up interviews (2014–2017), handgrip strength was measured using a digital dynamometer (Model T.K.K.5401 GRIP D; Takei Scientific Instruments Co., Ltd., Tokyo, Japan; Supplemental Method 3). As handgrip strength can be influenced by body measurements [20], and given the potential causal effect between baseline height and handgrip strength in our cohort [21], we also used grip strength (kg) per baseline height (m) as an outcome. Finally, consistent with previous studies [13,22], muscle weakness was defined as the lowest sex-specific quintile of handgrip strength in the study population (<21.5 kg in men and <13.4 kg in women).

### 2.5 Statistical analysis

Differences in baseline characteristics were tested by analysis of variance for continuous variables with a normal distribution, Kruskal-Wallis test for continuous variables without a normal distribution, and χ^2^ test for categorical variables.

Multivariable linear regression models were applied to assess the associations of UPF intake (sex-specific quintiles or a continuous variable) with absolute handgrip strength and handgrip strength relative to height. Results were shown as regression coefficients (β) and 95% confidence intervals (CIs). Linear trends were evaluated by using median values of each quintile as a continuous variable. Restricted cubic spline (RCS) regression models with three knots (10th, 50th, and 90th percentiles) were applied to assess the dose-response association.

Three multivariable models were fitted. Variance inflation factors (VIFs) were first used to test for multicollinearity among variables in the models and were all found to be within acceptable range (<10). In Model 1, we adjusted for age at the measurement of handgrip strength, sex, dialect group, educational level, alcohol frequency, smoking status, physical activity, sleep duration, total energy intake, caffeine, and body mass index (BMI). In Model 2, we additionally adjusted for comorbidities (diabetes, hypertension, cardiovascular diseases [coronary artery disease or stroke], cancer). In Model 3, we added the Alternative Healthy Eating Index (AHEI) [23] and the Vitamin C Equivalent Antioxidant Capacity (VCEAC) scores [24] to account for diet quality. Stratified analyses by age at baseline (≤55, >55 y) and sex (men, women) were conducted, and interactions were tested using the Wald test. In sensitivity analyses, we excluded participants with chronic conditions at baseline, reclassified whole-wheat bread and/or alcohol as UPFs, adjusted for covariates updated during the second follow-up, and studied grip strength as kg per weight (kg) or kg per BMI (kg/m^2^). Twenty-nine participants (0.2%) and 752 participants (5.5%) had missing values for VCEAC and BMI, respectively, and were excluded in the analyses that included these variables as covariates.

To further validate our results, we analyzed the association between UPF intake and muscle weakness. Multivariable logistic regression models were conducted to estimate odds ratios (ORs) and 95% CIs. Linear trends were tested by treating the median value in each quintile as a continuous variable. Using the Asian Working Group for Sarcopenia (AWGS) criteria (<28 kg for men and <18 kg for women) [25], we re-defined muscle weakness and repeated the analysis.

All analyses were performed using SAS version 9.4 (SAS Institute) and R software (version 4.4.2). Statistical significance was defined as two-sided *P* < 0.05.

## 3 Results

### 3.1 Participant characteristics

Of the 13,570 participants, the mean age was 53.2 ± 6.1 years at baseline, and 7,997 (58.9%) were women. After a median follow-up of 21.2 years, the mean age was 74.0 ± 6.2 years at the third follow-up. At baseline, participants with higher UPF consumption were less likely to smoke or drink alcohol, but more likely to have higher intake of energy, lower intake of caffeine, and lower scores of AHEI-2010 (*P* < 0.001; Table 1). Participants with muscle weakness were generally older, less educated, physically inactive, current smokers, never drinkers, and had a disease history (Supplemental Table S2).

**Table 1:** Baseline characteristics according to quintiles of ultra-processed food consumption among individuals in the Singapore Chinese Health Study.

	Sex-specific quintiles of ultra-processed food consumption^a^					
Characteristics^b^	Q1	Q2	Q3	Q4	Q5	*P* value
Weight ratio	1.2 (0.6−1.8)	3.5 (2.9−4.1)	6.2 (5.4−7.1)	10.9 (9.5−12.6)	19.7 (16.8−23.7)	–
Number of subjects	2713 (20.0)	2715 (20.0)	2714 (20.0)	2715 (20.0)	2713 (20.0)	–
Age at assessment of diet, years	53.5 ± 6.1	53.2 ± 6.0	53.0 ± 6.0	52.7 ± 5.9	53.4 ± 6.3	<0.001
Age at assessment of handgrip strength, years	74.5 ± 6.2	74.1 ± 6.1	73.8 ± 6.2	73.5 ± 6.1	74.2 ± 6.5	<0.001
Men	1114 (41.1)	1115 (41.1)	1115 (41.1)	1115 (41.1)	1114 (41.1)	–
Dialect group						0.005
Cantonese	1361 (50.2)	1446 (53.3)	1345 (49.6)	1324 (48.8)	1325 (48.8)	
Hokkien	1352 (49.8)	1269 (46.7)	1369 (50.4)	1391 (51.2)	1388 (51.2)	
Educational level						<0.001
No formal education	534 (19.7)	483 (17.8)	453 (16.7)	445 (16.4)	469 (17.3)	
Primary school	1292 (47.6)	1252 (46.1)	1127 (41.5)	1184 (43.6)	1187 (43.8)	
Secondary school or higher	887 (32.7)	980 (36.1)	1134 (41.8)	1086 (40.0)	1057 (39.0)	
Body mass index, kg/m^2^	23.5 ± 3.3	23.2 ± 3.3	23.1 ± 3.2	23.0 ± 3.2	22.7 ± 3.2	<0.001
Smoking status						<0.001
Never	1986 (73.2)	2088 (76.9)	2164 (79.7)	2159 (79.5)	2227 (82.1)	
Former	262 (9.7)	238 (8.8)	240 (8.8)	237 (8.7)	220 (8.1)	
Current	465 (17.1)	389 (14.3)	310 (11.4)	319 (11.8)	266 (9.8)	
Alcohol consumption						<0.001
None	2120 (78.1)	2153 (79.3)	2128 (78.4)	2183 (80.4)	2259 (83.3)	
Monthly	167 (6.2)	239 (8.8)	297 (10.9)	237 (8.7)	230 (8.5)	
Weekly	286 (10.5)	253 (9.3)	234 (8.6)	237 (8.7)	190 (7.0)	
Daily	140 (5.2)	70 (2.6)	55 (2.0)	58 (2.1)	34 (1.3)	
Physical activity						0.07
<0.5 h/wk	1773 (65.4)	1711 (63.0)	1680 (61.9)	1676 (61.7)	1684 (62.1)	
0.5−3.9 h/wk	574 (21.2)	636 (23.4)	667 (24.6)	636 (23.4)	634 (23.4)	
≥4 h/wk	366 (13.5)	368 (13.6)	367 (13.5)	403 (14.8)	395 (14.6)	
Sleep duration						0.01
<6 h/d	229 (8.4)	217 (8.0)	191 (7.0)	189 (7.0)	250 (9.2)	
6−8 h/d	2333 (86.0)	2337 (86.1)	2396 (88.3)	2384 (87.8)	2328 (85.8)	
>8 h/d	151 (5.6)	161 (5.9)	127 (4.7)	142 (5.2)	135 (5.0)	
History of hypertension	538 (19.8)	521 (19.2)	520 (19.2)	465 (17.1)	501 (18.5)	0.11
History of diabetes	145 (5.3)	126 (4.6)	105 (3.9)	139 (5.1)	113 (4.2)	0.05
History of cardiovascular disease	56 (2.1)	58 (2.1)	66 (2.4)	64 (2.4)	66 (2.4)	0.84
History of cancer	39 (1.4)	45 (1.7)	50 (1.8)	48 (1.8)	67 (2.5)	0.06
Dietary factors						
Total energy intake, kcal/d	1456.7 ± 485.7	1566.3 ± 510.9	1618.0 ± 512.8	1701.4 ± 546.6	1615.5 ± 488.3	<0.001
Caffeine	140.8 (88.3−227.3)	138.3 (88.3−226.6)	115.2 (82.4−224.3)	108.9 (61.4−222.4)	88.3 (32.9−144.3)	<0.001
AHEI-2010	51.4 ± 7.0	51.3 ± 6.7	51.1 ± 6.9	50.6 ± 7.6	49.9 ± 8.2	<0.001
VCEAC	253.3 (124.8−596.7)	269.5 (139.4−586.3)	257.7 (136.5−515.6)	268.4 (146.2−514.0)	210.4 (116.8−398.2)	<0.001

AHEI: Alternative Healthy Eating Index; VCEAC: Vitamin C Equivalent Antioxidant Capacity.

a Quintiles of the weight ratio of ultra-processed food intake in the total food consumed (%). Sex-specific cut-offs for quartiles of ultra-processed weight ratio were 1.9, 4.1, 7.3, and 13.4 in men and 2.7, 5.1, 8.8, and 15.5 in women.

b Values for categorical variables are given as numbers (%); values for continuous variables are given as mean ± SD or median (interquartile range).

### 3.2 Associations between UPF intake and handgrip strength

RCS curves showed that higher UPF intake was linearly associated with lower absolute handgrip strength and handgrip strength relative to height (Supplemental Figure S2). In Model 2, compared with participants in the lowest quintile of UPF intake, participants in the highest quintile had a reduction of 0.411 kg [95% CI, 0.148, 0.674] in absolute handgrip strength and a reduction of 0.229 kg/m (95% CI, 0.071, 0.387) in handgrip strength by height (Table 2). In Model 3, when additionally adjusted for AHEI-2010 and VCEAC, the risk estimates were slightly attenuated but still significant. Subgroup analyses showed the associations of UPF intake with handgrip strength were consistent across age and sex groups (all *P* for interaction ≥0.20) (Supplemental Table S3). The results remained similar in various sensitivity analyses (Supplemental Table S4–S5).

**Table 2:** β coefficients (95% confidence intervals) for associations of ultra-processed food intake with handgrip strength expressed in absolute and relative in the Singapore Chinese Health Study.

	Sex-specified quintiles of ultra-processed food consumption, %						
	Q1	Q2	Q3	Q4	Q5	*P* trend	Continuous^a^
Absolute handgrip strength, kg							
Model 1^b^	0 (ref)	−0.050 (-0.306, 0.206)	−0.082 (-0.340, 0.177)	−0.194 (-0.455, 0.068)	−0.397 (-0.661, -0.133)	<0.001	−0.195 (-0.304, -0.087)
Model 2^c^	0 (ref)	−0.058 (-0.313, 0.197)	−0.095 (-0.352, 0.163)	−0.193 (-0.453, 0.068)	−0.411 (-0.674, -0.148)	<0.001	−0.202 (-0.310, -0.094)
Model 3^d^	0 (ref)	−0.038 (-0.293, 0.217)	−0.058 (-0.316, 0.200)	−0.145 (-0.406, 0.116)	−0.338 (-0.603, -0.073)	0.005	−0.167 (-0.277, -0.058)
Handgrip strength relative to height, kg/m							
Model 1^b^	0 (ref)	−0.026 (-0.180, 0.128)	−0.030 (-0.185, 0.125)	−0.099 (-0.256, 0.058)	−0.220 (-0.379, -0.061)	0.002	−0.112 (-0.177, -0.046)
Model 2^c^	0 (ref)	−0.031 (-0.184, 0.122)	−0.038 (-0.193, 0.117)	−0.099 (-0.255, 0.057)	−0.229 (-0.387, -0.071)	0.002	−0.116 (-0.180, -0.051)
Model 3^d^	0 (ref)	−0.019 (-0.172, 0.134)	−0.017 (-0.172, 0.138)	−0.072 (-0.229, 0.084)	−0.188 (-0.347, -0.029)	0.009	−0.097 (-0.163, -0.032)

IQR: interquartile range.

a β coefficients for per increase of 10% in the proportion of ultra-processed food intake.

b Model 1 was adjusted for age at the measurement of handgrip strength (continuous, years), sex (men or women), total energy intake (continuous, kcal/day), dialect group (Cantonese or Hokkien), educational level (no formal education, primary school, or secondary school or higher), body mass index (continuous, kg/m^2^), smoking status (never, former, or current), alcohol frequency (never, monthly, weekly, or daily), physical activity (<0.5, 0.5–3.9, or ≥4.0 h/week), sleep duration (<6, 6–8, or >8 h/day), and caffeine (continuous, mg/day).

c Model 2 was adjusted for the variables listed in Model 1 + history of hypertension (yes or no), history of diabetes (yes or no), history of cardiovascular disease (yes or no), and history of cancer (yes or no).

d 29 participants were not included in these analyses because of missing values of Vitamin C Equivalent Antioxidant Capacity. Model 3 was adjusted for the variables listed in Model 2 + Alternative Healthy Eating Index-2010 (continuous), and Vitamin C Equivalent Antioxidant Capacity (continuous).

Table 3 shows the association between UPF intake and the risk of muscle weakness. In Model 2, compared with participants in the first quintile of UPF intake, the adjusted OR (95% CI) for participants in the fifth quintile was 1.32 (1.15, 1.53). Adjusting for AHEI-2010 and VCEAC scores did not change the results. The positive association between UPF intake and muscle weakness was also observed when using the cut-off value proposed by the AWGS (Supplemental Table S6).

**Table 3:** Odds ratios (95% confidence intervals) for associations of ultra-processed food intake with muscle weakness in the Singapore Chinese Health Study.^a^

	Sex-specified quintiles of ultra-processed food consumption, %						
	Q1	Q2	Q3	Q4	Q5	*P* trend	Continuous^b^
Muscle weakness							
Model 1^c^	1 (ref)	1.07 (0.93, 1.23)	1.04 (0.90, 1.21)	1.14 (0.99, 1.32)	1.31 (1.14, 1.51)	<0.001	1.11 (1.05, 1.18)
Model 2^d^	1 (ref)	1.07 (0.93, 1.24)	1.05 (0.91, 1.21)	1.14 (0.99, 1.32)	1.32 (1.15, 1.53)	<0.001	1.12 (1.06, 1.18)
Model 3^e^	1 (ref)	1.07 (0.93, 1.23)	1.03 (0.89, 1.20)	1.12 (0.97, 1.30)	1.27 (1.10, 1.47)	<0.001	1.10 (1.04, 1.16)

IQR: interquartile range.

a Muscle weakness was defined as the lowest sex-specific quintile of handgrip strength in the study population (<21.5 kg in men and < 13.4 kg in women).

b Odds ratios for per increase of 10% in the proportion of ultra-processed food intake.

c Model 1 was adjusted for age at the measurement of handgrip strength (continuous, years), sex (men or women), total energy intake (continuous, kcal/day), dialect group (Cantonese or Hokkien), educational level (no formal education, primary school, or secondary school or higher), body mass index (continuous, kg/m^2^), smoking status (never, former, or current), alcohol frequency (never, monthly, weekly, or daily), physical activity (<0.5, 0.5–3.9, or ≥4.0 h/week), sleep duration (<6, 6–8, or >8 h/day), and caffeine (continuous, mg/day).

d Model 2 was adjusted for the variables listed in Model 1 + history of hypertension (yes or no), history of diabetes (yes or no), history of cardiovascular disease (yes or no), and history of cancer (yes or no).

e 29 participants were not included in these analyses because of missing values of Vitamin C Equivalent Antioxidant Capacity. Model 3 was adjusted for the variables listed in Model 2 + Alternative Healthy Eating Index-2010 (continuous), and Vitamin C Equivalent Antioxidant Capacity (continuous).

## 4 Discussion

In this large prospective study, higher UPF intake at midlife was associated with lower handgrip strength and an increased risk of muscle weakness in late life. These associations remained significant after adjusting diet quality.

Our finding concurred with one study conducted in Tianjin, China, which revealed that higher UPF intake was associated with faster handgrip strength decline [13]. Three studies also showed higher UPF intake was related to lower muscle mass [[10], [11], [12]]. Together, these studies and ours provided evidence for the association between UPF and sarcopenia, a condition characterized by loss of muscle mass and strength in older adults [25].

Furthermore, the association between UPF intake and handgrip strength was still significant after adjusting for AHEI-2010 and VCEAC in our study. The aforementioned study involving Chinese adults also observed a significant association even after adjusting for diet factors [13]. Hence, UPFs may have biologically adverse effects on muscle health. Chemicals added to UPFs (e.g., emulsifiers), formed during processing (e.g., advanced glycation end products), or migrating from packaging (e.g., phthalates) can cause inflammation and lower serum androgens, potentially reducing muscle strength [[26], [27], [28], [29], [30]].

The study’s strength includes its long follow-up, prospective design, and relatively large sample size, but several limitations should be considered. First, misclassification of UPF intake could occur since the FFQ used was not tailored to the Nova classification, and changes during follow-up were not captured due to one-time diet and handgrip strength assessments. Additionally, the evolving types and quantities of UPFs in Asia may lead to inaccuracies and weaken the causal interpretations. Second, baseline exclusion of participants with chronic diseases mitigated pre-existing muscle weakness, but the lack of initial grip strength measurements leaves some uncertainty. Third, survivorship bias is also possible, as participants who had died or become disabled by the third follow-up were excluded. Nonetheless, the weight proportion of UPF intake was similar between study participants and non-participants of the follow-up (median: 6.2% vs 6.0%).

## Conclusions

We found that higher UPF intake at mild life was associated with lower handgrip strength at late life in an Asian population, and this was not explained by overall diet quality.

## CRediT authorship contribution statement

Drs Pan and Koh had full access to all of the data in the study and take responsibility for the integrity of the data and the accuracy of the data analysis.

Concept and design: Yue Li, An Pan, and Woon-Puay Koh.

Statistical analysis: Yue Li and Kevin Y. Chua.

Drafting of the manuscript: Yue Li.

Critical revision of the manuscript for important intellectual content: All authors.

Supervision: An Pan and Woon-Puay Koh.

## Additional contributions

We thank Ms. Siew-Hong Low of the National University of Singapore for supervising the fieldwork of the Singapore Chinese Health Study.

## Declaration of Generative AI and AI-assisted technologies in the writing process

We have not used any AI.

## Funding

This work was supported by the Singapore National Medical Research Council [NMRC/CSA/0055/2013 and the Singapore Strategic Cohorts Consortium Award (23-1034-A0001)]; the United States National Institutes of Health [UM1 CA182876, and R01CA144034]; and the Saw Swee Hock School of Public Health, National University of Singapore. Dr Pan was funded by grants from the National Natural Science Foundation of China (82325043). Dr Koh was supported by the National Medical Research Council, Singapore [CSA-SI (MOH-000434)]. The funding agencies play no role in the design or conduct of the study; collection, management, analysis, or interpretation of the data; preparation, review, or approval of the manuscript; or decision to submit the manuscript for publication.

## Data sharing

Data described in the article, code book, and analytic code will be made available upon reasonable request.

## Declaration of competing interest

The authors declare that they have no known competing financial interests or personal relationships that could have appeared to influence the work reported in this paper.
