# Supplementary material for: Association between consumption of ultra-processed food at midlife and handgrip strength at late life: The Singapore Chinese Health Study
Source: J Nutr Health Aging. 2025 Jul 18;29(9):100634. doi: 10.1016/j.jnha.2025.100634 (PMC12378926; doi:10.1016/j.jnha.2025.100634)
Supplement: Supplementary file 1 [file mmc1.docx]

**Association between ultra-processed food consumption at midlife and handgrip strength at late life: the Singapore Chinese Health Study**

**Online Supplementary Material**

**Supplemental Method 1:** The detailed information of FFQ

**Supplemental Method 2:** The assessment of covariates

**Supplemental Method 3:** The measurement of handgrip strength

**Supplemental Table S1.** Food products considered as ultra-processed foods according to the NOVA classification

**Supplemental Table S2.** Baseline characteristics according to the status of muscle weakness among individuals in the Singapore Chinese Health Study

**Supplemental Table S3.** β coefficients (95% confidence intervals) for associations between ultra-processed food intake and handgrip strength expressed in absolute and relative terms in different strata in the Singapore Chinese Health Study

**Supplemental Table S4.** Sensitivity analysis for associations of ultra-processed food intake with handgrip strength expressed in absolute and relative terms in the Singapore Chinese Health Study

**Supplemental Table S5**. β coefficients (95% confidence intervals) for associations of ultra-processed food intake with handgrip strength expressed in two relative terms in the Singapore Chinese Health Study

**Supplemental Table S6.** Odds ratios (95% confidence intervals) for associations of ultra-processed food intake with muscle weakness according to the AWGS cut-off in the Singapore Chinese Health Study

**Supplemental Figure S1.** Selection of study participants

**Supplemental Figure S2.** Restricted cubic spline analysis of ultra-processed food consumption and handgrip strength expressed in absolute and relative terms in the Singapore Chinese Health Study

**Supplemental Method 1:** **The detailed information of FFQ**

For each food item, participants were asked to choose from eight intake frequencies (ranging from “never or hardly ever” to “two or more times a day”) and three serving sizes (small, medium, or large). The daily intakes of energy and nutrients were calculated based on the Singapore Food Composition Database, which was specifically developed for this cohort.(1) The FFQ was subsequently validated against two 24-h recalls within a subcohort of participants from SCHS, with correlation coefficients of selected nutrients ranging from 0.24 to 0.79, which were considered comparable to calibration studies of other populations.(1)

**Supplemental Method 2: The assessment of covariates**

At baseline, trained investigators collected data on participants' demographic characteristics (age, sex, dialect group, and education), lifestyle factors (alcohol frequency, smoking status, physical activity, and sleep duration), anthropometric data (weight and height), and medical history (coronary artery disease, stroke, hypertension, diabetes, and cancer) using a structured questionnaire. The history of cancer was also ascertained through the nationwide Singapore Cancer Registry. Hours per week spent on moderate activities (e.g. bowling and brisk walking), strenuous sports (e.g. jogging and tennis), and vigorous work (e.g. moving heavy furniture and shoveling) during the last year were collected and categorized into <0.5, 0.5 to 3.9, or ≥4.0 hours/week.(2, 3) Body mass index (BMI) was derived from body weight (kg) divided by the square of the height (m). The daily caffeine intake was calculated by summing up the caffeine from beverages (e.g. coffee or tea) and food items (e.g. chocolate).(1) The Alternative Healthy Eating Index (AHEI)-2010 score was calculated from 10 components that included vegetables, fruits, whole grains, sugar-sweetened beverages and fruit juice, red meat, long-chain n–3 polyunsaturated fatty acids (PUFAs), nuts and legumes, sodium, and alcohol; trans-fat was not included due to the lack of information on consumption level in our cohort and the very low overall consumption known to be the case in Singapore.(4) The Vitamin C Equivalent Antioxidant Capacity (VCEAC) was calculated using the database of Vitamin C Equivalents (VCE) and considered the intake levels of 12 antioxidants including 5 categories of flavonoids, 5 categories of carotenoids, vitamin C, and vitamin E.(5) During the second follow-up, we updated information regarding lifestyle factors (alcohol frequency, smoking status, and physical activity), anthropometric data (weight and height), and medical history (coronary artery disease, stroke, hypertension, and diabetes). Physical activity at the second follow-up was measured as hours per day or week spent on moderate activities, strenuous sports, and vigorous work during the last year, and categorized into <0.5, 0.5 to 3.9, or ≥4.0 hours/week.(2, 3)

**Supplemental Method 3: The measurement of handgrip strength**

Within the measurement, participants clasped the digital dynamometer using full force while standing up with arms let down naturally. Digital readings were recorded to the nearest 0.1 kg. Each participant recorded two grip strengths with each hand, beginning with the right and in alternate order, and only the highest grip measured from each hand was used. The overall handgrip strength was calculated as the mean of the readings from two hands.

**Supplemental Table S1.** **Food products considered as ultra-processed foods according to the NOVA classification.**

| Ultra-processed food group | Examples of food |
| --- | --- |
| Beverages | Soft drinks, Milo, Ovaltine, and Horlicks |
| Dairy based products | Yakult, Vitagen, ice cream, and frozen yogurt |
| Cereals and starchy foods^a^ | Hot oats or other hot cereals, cornflakes or other cold cereals, white sliced bread, and hotcake |
| Sugary products | Crackers and biscuits, western cakes, and coconut desserts |
| Savory snacks | Hamburger and cheeseburger, French fries, sandwiches, pizza, and baked buns with processed meat |
| Meat and eggs | Deep-fried chicken, sausage, ham, hot dogs, luncheon meat, deep-fried fish, and preserved eggs |
| Spread and sweetener | Margarine, peanut butter, and artificial sweetener |

^a^ Considering whole-wheat bread was associated with a lower risk of ischemic heart disease mortality in a previous study conducted in the Singapore Chinese Health Study, we did not include whole-wheat bread in our primary analysis.

**Supplemental Table S2.** **Baseline characteristics according to the status of muscle weakness among individuals in the Singapore Chinese Health Study.**

|  | Muscle weakness | |  |
| --- | --- | --- | --- |
| Characteristics^a^ | No (n = 10858) | Yes (n = 2712) | *P* value |
| Age at assessment of diet, years | 52.3 ± 5.6 | 56.5 ± 6.6 | <0.001 |
| Age at assessment of handgrip strength, years | 73.1 ± 5.8 | 77.5 ± 6.6 | <0.001 |
| Men | 4454 (41.0) | 1119 (41.3) | - |
| Dialect group |  |  | <0.001 |
| Cantonese | 5266 (48.5) | 1535 (56.6) |  |
| Hokkien | 5592 (51.5) | 1177 (43.4) |  |
| Educational level |  |  | <0.001 |
| No formal education | 1756 (16.2) | 628 (23.2) |  |
| Primary school | 4738 (43.6) | 1304 (48.1) |  |
| Secondary school or higher | 4364 (40.2) | 780 (28.8) |  |
| Body mass index, kg/m^2^ | 23.1 ± 3.2 | 23.0 ± 3.3 | 0.02 |
| Smoking status |  |  | <0.001 |
| Never | 8583 (79.0) | 2041 (75.3) |  |
| Former | 934 (8.6) | 263 (9.7) |  |
| Current | 1341 (12.4) | 408 (15.0) |  |
| Alcohol consumption |  |  | <0.001 |
| None | 8605 (79.3) | 2238 (82.5) |  |
| Monthly | 982 (9.0) | 188 (6.9) |  |
| Weekly | 983 (9.1) | 217 (8.0) |  |
| Daily | 288 (2.7) | 69 (2.5) |  |
| Physical activity |  |  | <0.001 |
| <0.5 h/wk | 6730 (62.0) | 1794 (66.2) |  |
| 0.5-3.9 h/wk | 2596 (23.9) | 551 (20.3) |  |
| ≥4 h/wk | 1532 (14.1) | 367 (13.5) |  |
| Sleep duration |  |  | <0.001 |
| <6 h/d | 804 (7.4) | 272 (10.0) |  |
| 6-8 h/d | 9509 (87.6) | 2269 (83.7) |  |
| >8 h/d | 545 (5.0) | 171 (6.3) |  |
| History of hypertension | 1913 (17.6) | 632 (23.3) | <0.001 |
| History of diabetes | 427 (3.9) | 201 (7.4) | <0.001 |
| History of cardiovascular disease | 214 (2.0) | 96 (3.5) | <0.001 |
| History of cancer | 195 (1.8) | 54 (2.0) | 0.50 |

^a^ Values for categorical variables are given as numbers (%); values for continuous variables are given as mean ± SD or median (interquartile range).

**Supplemental Table S3. β coefficients (95% confidence intervals) for associations between ultra-processed food intake and** **handgrip strength expressed in absolute and relative terms in different strata in the Singapore Chinese Health Study.^a^**

|  | Sex-specific quintiles of ultra-processed food consumption, % | | | | |  |  |
| --- | --- | --- | --- | --- | --- | --- | --- |
|  | Q1 | Q2 | Q3 | Q4 | Q5 | *P* trend | *P* for interaction |
| Absolute handgrip strength, kg |  |  |  |  |  |  |  |
| Age at baseline, years |  |  |  |  |  |  |  |
| ≤55 (n = 8829) | 0 (ref) | -0.133 (-0.460, 0.193) | -0.157 (-0.485, 0.171) | -0.197 (-0.528, 0.133) | -0.340 (-0.680, 0.001) | 0.06 | 0.56 |
| >55 (n = 4741) | 0 (ref) | 0.145 (-0.257, 0.546) | 0.171 (-0.242, 0.584) | -0.049 (-0.469, 0.372) | -0.375 (-0.790, 0.040) | 0.01 |  |
| Sex |  |  |  |  |  |  |  |
| Men (n = 5573) | 0 (ref) | 0.120 (-0.360, 0.601) | 0.204 (-0.284, 0.692) | 0.009 (-0.484, 0.502) | -0.103 (-0.606, 0.401) | 0.40 | 0.20 |
| Women (n = 7997) | 0 (ref) | -0.103 (-0.372, 0.165) | -0.183 (-0.455, 0.089) | -0.229 (-0.505, 0.047) | -0.480 (-0.760, -0.201) | <0.001 |  |
| Handgrip strength relative to height, kg/m | |  |  |  |  |  |  |
| Age at baseline, years |  |  |  |  |  |  |  |
| ≤55 (n = 8829) | 0 (ref) | -0.081 (-0.277, 0.114) | -0.085 (-0.281, 0.111) | -0.109 (-0.307, 0.088) | -0.188 (-0.391, 0.016) | 0.09 | 0.53 |
| >55 (n = 4741) | 0 (ref) | 0.099 (-0.145, 0.342) | 0.133 (-0.117, 0.383) | -0.008 (-0.263, 0.247) | -0.209 (-0.460, 0.043) | 0.02 |  |
| Sex |  |  |  |  |  |  |  |
| Men (n = 5573) | 0 (ref) | 0.083 (-0.198, 0.364) | 0.173 (-0.113, 0.458) | 0.030 (-0.258, 0.318) | -0.058 (-0.353, 0.236) | 0.36 | 0.34 |
| Women (n = 7997) | 0 (ref) | -0.066 (-0.234, 0.102) | -0.120 (-0.290, 0.050) | -0.134 (-0.307, 0.038) | -0.267 (-0.442, -0.092) | 0.003 |  |

^a^ 29 participants were not included in these analyses because of missing values of Vitamin C Equivalent Antioxidant Capacity. Adjusted for age at the measurement of handgrip strength, sex, total energy intake, dialect group, educational level, body mass index, smoking status, alcohol consumption, physical activity, sleep duration, caffeine, history of hypertension, history of diabetes, history of cardiovascular disease, history of cancer, Alternative Healthy Eating Index-2010, and Vitamin C Equivalent Antioxidant Capacity. The strata variable was not included in the model when stratifying by itself.

**Supplemental Table S4. Sensitivity analysis for associations of ultra-processed food intake with handgrip strength expressed in absolute and relative terms in the Singapore Chinese Health Study.^a^**

|  | Absolute handgrip strength, kg | Handgrip strength relative to height, kg/m |
| --- | --- | --- |
|  | β coefficients (95% CI)^b^ | β coefficients (95% CI)^b^ |
| Excluded those with hypertension, diabetes, cardiovascular disease or cancer at baseline | -0.156 (-0.280, -0.031) | -0.093 (-0.168, -0.019) |
| Included whole-wheat bread in the total UPF definition | -0.157 (-0.266, -0.049) | -0.090 (-0.155, -0.025) |
| Included distilled alcohol in the total UPF definition | -0.168 (-0.277, -0.059) | -0.098 (-0.163, -0.032) |
| Included whole-wheat bread and distilled alcohol in the total UPF definition | -0.158 (-0.266, -0.049) | -0.091 (-0.156, -0.026) |
| Further adjusted updated variables collected at the second follow-up^c^ | -0.141 (-0.253, -0.030) | -0.082 (-0.149, -0.015) |

CI: confidence intervals; UPF: ultra-processed food.

^a^29 participants were not included in these analyses because of missing values of Vitamin C Equivalent Antioxidant Capacity. Adjusted for age at the measurement of handgrip strength (continuous, years), sex (men or women), total energy intake (continuous, kcal/day), dialect group (Cantonese or Hokkien), educational level (no formal education, primary school, or secondary school or higher), body mass index (continuous, kg/m^2^), smoking status (never, former, or current), alcohol frequency (never, monthly, weekly, or daily), physical activity (<0.5, 0.5 to 3.9, or ≥4.0 hours/week), sleep duration (<6, 6 to 8, or >8 h/day), caffeine (continuous, mg/day), history of hypertension (yes or no), history of diabetes (yes or no), history of cardiovascular disease (yes or no), history of cancer (yes or no), Alternative Healthy Eating Index-2010 (continuous), and Vitamin C Equivalent Antioxidant Capacity (continuous).

^b^ β coefficients for per increase of 10% in the proportion of ultra-processed food intake.

^c^ 752 participants were further excluded in this analysis because of missing values of BMI at the second follow-up. Further adjusted for the second follow-up variables of physical activity (<0.5, 0.5 to 3.9, or ≥4.0 hours/week), body mass index (continuous, kg/m^2^), smoking status (never, former, or current), alcohol frequency (never, monthly, weekly, or daily), history of hypertension (yes or no), history of diabetes (yes or no), and history of cardiovascular disease (yes or no).

**Supplemental Table S5. β coefficients (95% confidence intervals) for associations of ultra-processed food intake with** **handgrip strength** **expressed in two relative terms in the Singapore Chinese Health Study.^a^**

|  | Sex-specific quintiles of ultra-processed food consumption, % | | | | |  | |  |
| --- | --- | --- | --- | --- | --- | --- | --- | --- |
|  | Q1 | Q2 | Q3 | Q4 | Q5 | | *P* trend | Continuous^b^ |
| Handgrip strength relative to weight, kg/kg | |  |  |  |  | |  |  |
| Model^c^ | 0 (ref) | 0.000 (-0.004, 0.005) | 0.001 (-0.003, 0.006) | -0.004 (-0.008, 0.001) | -0.006 (-0.011, -0.001) | | <0.001 | -0.003 (-0.005, -0.001) |
| Model plus AHEI-2010 plus VCEAC^d^ | 0 (ref) | 0.000 (-0.004, 0.005) | 0.002 (-0.003, 0.006) | -0.003 (-0.008, 0.001) | -0.005 (-0.010, -0.001) | | 0.003 | -0.003 (-0.005, -0.001) |
| Handgrip strength relative to BMI, kg/(kg/m^2^) | |  |  |  |  | |  |  |
| Model^c^ | 0 (ref) | -0.001 (-0.013, 0.011) | -0.001 (-0.014, 0.011) | -0.014 (-0.026, -0.001) | -0.021 (-0.034, -0.009) | | <0.001 | -0.011 (-0.016, -0.006) |
| Model plus AHEI-2010 plus VCEAC^d^ | 0 (ref) | 0.000 (-0.012, 0.012) | 0.000 (-0.013, 0.012) | -0.012 (-0.025, 0.000) | -0.019 (-0.032, -0.006) | | <0.001 | -0.010 (-0.015, -0.004) |

AHEI: Alternative Healthy Eating Index; VCEAC: Vitamin C Equivalent Antioxidant Capacity.

^a^ Including 13006 participants considering the exclusion of missing weight or BMI during the third follow-up.

^b^ β coefficients for per increase of 10% in the proportion of ultra-processed food intake.

^c^ Adjusted for age at the measurement of handgrip strength (continuous, years), sex (men or women), total energy intake (continuous, kcal/day), dialect group (Cantonese or Hokkien), educational level (no formal education, primary school, or secondary school or higher), body mass index (continuous, kg/m^2^), smoking status (never, former, or current), alcohol frequency (never, monthly, weekly, or daily), physical activity (<0.5, 0.5 to 3.9, or ≥4.0 hours/week), sleep duration (<6, 6 to 8, or >8 h/day), caffeine (continuous, mg/day), history of hypertension (yes or no), history of diabetes (yes or no), history of cardiovascular disease (yes or no), and history of cancer (yes or no).

^d^ 29 participants were not included in these analyses because of missing values of Vitamin C Equivalent Antioxidant Capacity.

**Supplemental Table S6. Odds ratios (95% confidence intervals) for associations of ultra-processed food intake with** **muscle weakness according to the AWGS cut-off in the Singapore Chinese Health Study.^a^**

|  | Sex-specified quintiles of ultra-processed food consumption, % | | | | |  |  |
| --- | --- | --- | --- | --- | --- | --- | --- |
|  | Q1 | Q2 | Q3 | Q4 | Q5 | *P* trend | Continuous^b^ |
| Model^c^ | 1 (ref) | 0.99 (0.88, 1.11) | 1.03 (0.91, 1.16) | 1.11 (0.98, 1.25) | 1.14 (1.01, 1.29) | 0.006 | 1.08 (1.03, 1.14) |
| Model plus AHEI-2010 plus VCEAC^d^ | 1 (ref) | 0.98 (0.88, 1.10) | 1.02 (0.91, 1.15) | 1.10 (0.97, 1.23) | 1.12 (0.99, 1.27) | 0.01 | 1.08 (1.02, 1.13) |

AWGS: Asian Working Group for Sarcopenia; AHEI: Alternative Healthy Eating Index; VCEAC: Vitamin C Equivalent Antioxidant Capacity.

^a^ Muscle weakness was defined as < 28 kg in men and < 18 kg in women according to the AWGS 2019 consensus.

^b^ Odds ratios for per increase of 10% in the proportion of ultra-processed food intake.

^c^ Adjusted for age at the measurement of handgrip strength (continuous, years), sex (men or women), total energy intake (continuous, kcal/day), dialect group (Cantonese or Hokkien), educational level (no formal education, primary school, or secondary school or higher), body mass index (continuous, kg/m^2^), smoking status (never, former, or current), alcohol frequency (never, monthly, weekly, or daily), physical activity (<0.5, 0.5 to 3.9, or ≥4.0 hours/week), sleep duration (<6, 6 to 8, or >8 h/day), caffeine (continuous, mg/day), history of hypertension (yes or no), history of diabetes (yes or no), history of cardiovascular disease (yes or no), and history of cancer (yes or no).

^d^ 29 participants were not included in these analyses because of missing values of Vitamin C Equivalent Antioxidant Capacity.

**Supplemental Figure S1. Selection of study participants.**

Participants recruited at baseline with FFQ (n = 63257)

Surviving participants re-contacted at third follow-up interviews with measurement of handgrip strength (n = 13789)

Excluded (n = 219):

Extreme energy intake (men: total energy<700; >3700 kcal/day; women: total energy<600; >3000 kcal/day) (n = 168);

Without self-reported height at baseline (n = 51)

Participants included in the present study (n = 13570)

**Supplemental Figure S2. Restricted cubic spline analysis of ultra-processed food consumption and handgrip strength expressed in absolute and relative terms in the Singapore Chinese Health Study.**


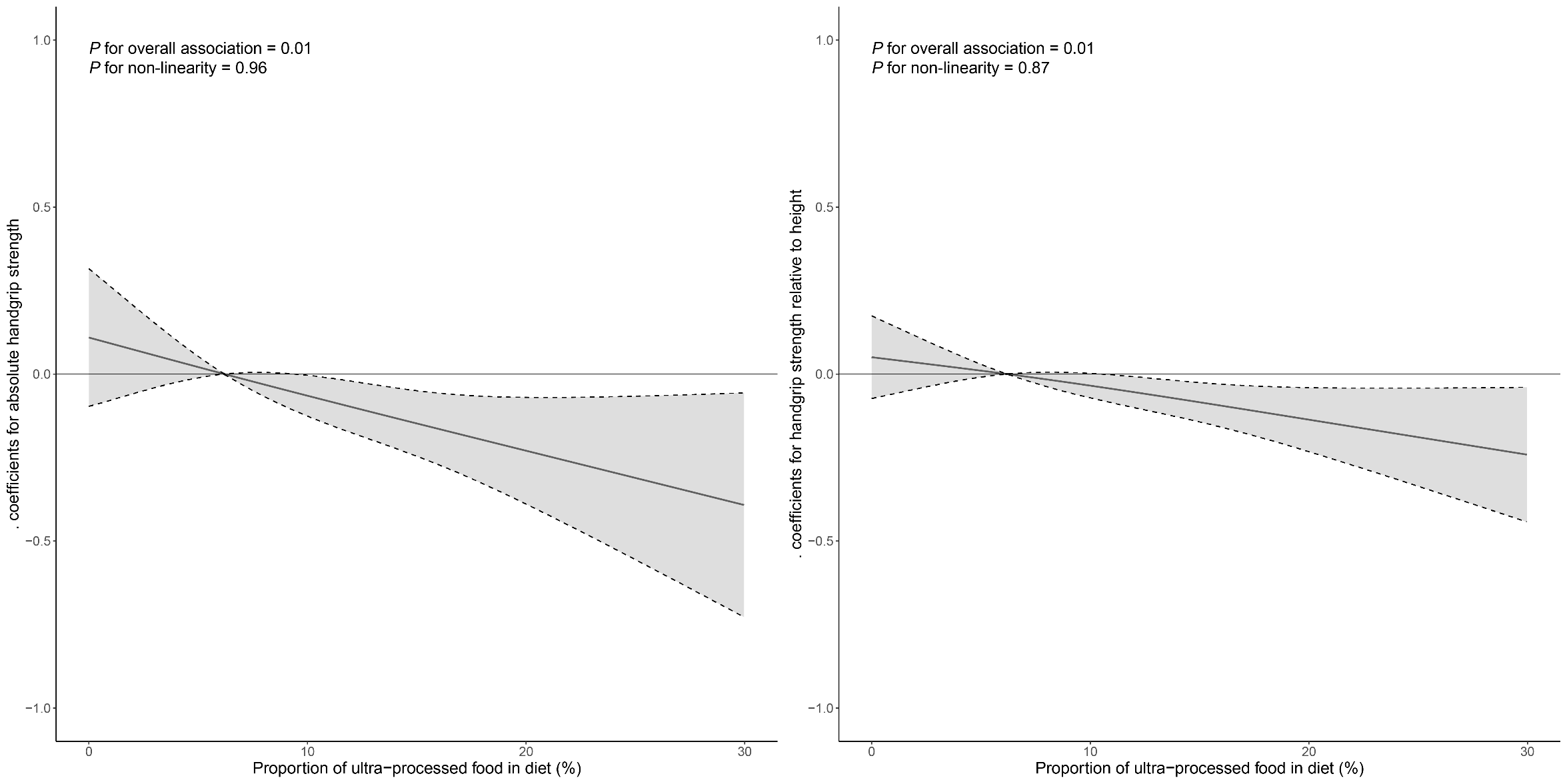


29 participants were not included in these analyses because of missing values of Vitamin C Equivalent Antioxidant Capacity. HRs with 95% confidence intervals (CIs) were calculated based on the multivariable model adjusted for age at the measurement of handgrip strength (continuous, years), sex (men or women), total energy intake (continuous, kcal/day), dialect group (Cantonese or Hokkien), educational level (no formal education, primary school, or secondary school or higher), body mass index (continuous, kg/m^2^), smoking status (never, former, or current), alcohol frequency (never, monthly, weekly, or daily), physical activity (<0.5, 0.5 to 3.9, or ≥4.0 hours/week), sleep duration (<6, 6 to 8, or >8 h/day), caffeine (continuous, mg/day), history of hypertension (yes or no), history of diabetes (yes or no), history of cardiovascular disease (yes or no), history of cancer (yes or no), Alternative Healthy Eating Index-2010 (continuous), and Vitamin C Equivalent Antioxidant Capacity (continuous). The reference values for HRs were set as 6.2% (the median value of the proportion of ultra-processed food intake). Three knots were located at the 10th, 50th, and 90th percentiles of exposure. The gray zones indicated 95% CIs.

**Reference**

1. Hankin JH, Stram DO, Arakawa K, Park S, Low SH, Lee HP, et al. Singapore Chinese Health Study: development, validation, and calibration of the quantitative food frequency questionnaire. Nutrition and cancer. 2001;39(2):187-95.

2. Zhang J-J, Ye Y-X, Dorajoo R, Khor C-C, Chang X-L, Yu H-C, et al. APOE Genotype Modifies the Association between Midlife Adherence to the Planetary Healthy Diet and Cognitive Function in Later Life among Chinese Adults in Singapore. The Journal of Nutrition. 2024;154(1):252-60.

3. Ye YX, Geng TT, Zhou YF, He P, Zhang JJ, Liu G, et al. Adherence to a Planetary Health Diet, Environmental Impacts, and Mortality in Chinese Adults. JAMA Netw Open. 2023;6(10):e2339468.

4. Wu J, Song X, Chen G-C, Neelakantan N, van Dam RM, Feng L, et al. Dietary pattern in midlife and cognitive impairment in late life: a prospective study in Chinese adults. The American Journal of Clinical Nutrition. 2019;110(4):912-20.

5. Floegel A, Kim DO, Chung SJ, Song WO, Fernandez ML, Bruno RS, et al. Development and validation of an algorithm to establish a total antioxidant capacity database of the US diet. Int J Food Sci Nutr. 2010;61(6):600-23.
